# MicroRNA-206 attenuates the growth and angiogenesis in non-small cell lung cancer cells by blocking the 14-3-3ζ/STAT3/HIF-1α/VEGF signaling

**DOI:** 10.18632/oncotarget.12972

**Published:** 2016-10-28

**Authors:** Dong Xue, Ye Yang, Yawei Liu, Peiwen Wang, Yi Dai, Qinqiang Liu, Lijun Chen, Jian Shen, Huanyu Ju, Yuan Li, Zhenguo Tan

**Affiliations:** ^1^ Department of Surgery, The Second Affiliated Hospital, Nanjing Medical University, Nanjing 211166, China; ^2^ Department of Nutrition And Food Hygiene, School Of Public Health, Nanjing Medical University, Nanjing 211166, China

**Keywords:** lung cancer, microRNA-206, 14-3-3ζ, angiogenesis, signal transduction

## Abstract

Non-small cell lung cancer (NSCLC) is the leading cause of cancer-related mortality worldwide. Angiogenesis is the major hallmark in NSCLC. So, further elucidation of molecular mechanisms underlying the angiogenesis of NSCLC is urgently needed. Here, we found that microRNA-206 (miR-206) decreased the angiogenic ability in NSCLC via inhibiting the 14-3-3ζ/STAT3/HIF-1α/VEGF pathway. Briefly, 14-3-3ζ bond with phosphorylated-STAT3, and in turn, elevated the expression of HIF-1α. Then, by enhancing the recruitment of HIF-1α to *VEGF* promoter, 14-3-3ζ increased the angiogenesis. However, miR-206 decreased the angiogenesis by targeting 14-3-3ζ, and inhibiting the STAT3/HIF-1α/VEGF pathway. In NSCLC cell xenograft model, either overexpression of miR-206 or inhibition of 14-3-3ζ inhibited the STAT3/HIF-1α/VEGF pathway and decreased the tumor growth and angiogenesis. Furthermore, there was a negative correlation between miR-206 and 14-3-3ζ in NSCLC specimens. NSCLC patients with low expressions of miR-206 but high expressions of 14-3-3ζ had the worst survival. Collectively, our findings provided the underlying mechanisms of miR-206/14-3-3ζ in tumor growth and angiogenesis, and implicated miR-206 and 14-3-3ζ as potential therapeutic targets for NSCLC.

## INTRODUCTION

Lung cancer is the leading cause of cancer-related mortality worldwide [1]. The NSCLC, including non-squamous carcinomas and the squamous carcinomas, accounts for approximately 80% of lung cancer [2]. Up to date, the long-term outcome of NSCLC is poor because of the high metastatic ratio [3]. The lymphatic metastasis represents a major shift in NSCLC biology [4], nevertheless, the hematogenous metastasis, which spreads cancerous cell to bone and/or brain, plays a key role in the progression of NSCLC [5, 6]. In fact, the cancer-related angiogenesis has been proposed as the major hallmark in NSCLC [7]. Consequently, further elucidation of mechanisms underlying the angiogenesis of NSCLC is urgently needed.

The 14-3-3 proteins are a family of approximately 28 to 33 kDa acidic polypeptides, which have seven mammalian isoforms (α/β, γ, σ, ε, ζ, η, and θ/τ). Up to date, six members of 14-3-3 family proteins (α/β, γ, σ, ε, θ/τ, and ζ) have been identified to be positively expressed and play roles in the NSCLC [8]. Especially, 14-3-3ζ, which complexes with Hsp27 or β-catenin, leading to the epithelial to mesenchymal transition, metastasis, and has been implicated as a prognostic and therapeutic target [9–11]. However, the functions of 14-3-3ζ in the angiogenesis of NSCLC, and the potential molecular mechanisms underlying remain unclear.

The miRNAs are a group of small non-coding RNAs, which inhibit the target genes by binding to their 3′-UTR [12–15]. MiR-206 is an important tumor suppressor in lung cancer. It suppresses the NSCLC growth, metastasis, and cisplatin resistance via targeting c-Met, Bcl2, cyclinD1, and Sox9 [16–18]. Here, we found a miR-206 binding site in 3′-UTR of *14-3-3ζ* mRNA. So, our present study aimed to indicate the potential signal transduction initiated by miR-206/14-3-3ζ, and to provide a better understanding of miR-206- and/or 14-3-3ζ-caused angiogenesis in NSCLC.

## RESULTS

### Effects of 14-3-3ζ on the angiogenic ability in NSCLC cells

**Figure 1:**
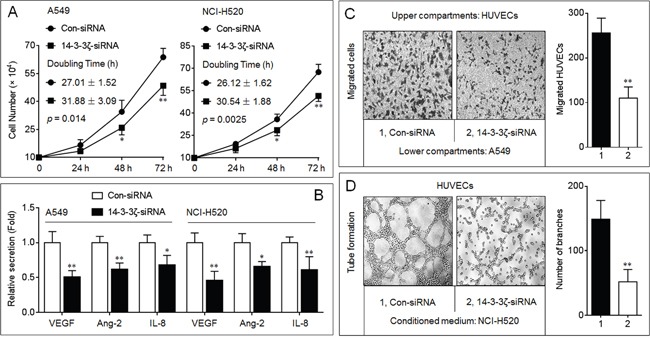
Effects of 14-3-3ζ on the angiogenic ability in NSCLC cells **A.** Growth kinetics analyses in triplicate in A549 and NCI-H520 cells with different 14-3-3ζ levels. The difference between two growth curves was significant (in A549, p = 0.014, while in NCI-H520, p = 0.0025). **B.** ELISA analyses in triplicate of the secretions of VEGF, angiopoietin-2 (Ang-2), and IL-8. The values of chemotactic factors secreted by Con-siRNA-transfected cells were defined as 1 fold. **C.** Endothelial cells recruitment assay analyses of the effects of 14-3-3ζ on A549-induced recruitment of HUVECs. **D.** Capillary tube formation assay analyses of the effects of 14-3-3ζ on angiogenic ability in NCI-H520 cells. * *p* < 0.05 and ** *p* < 0.01 compared with the Con-siRNA-transfected NSCLC cells.

As shown in Supplementary Figure S1, the expressions of 14-3-3ζ were increased in four NSCLC cell lines compared to that in BEAS-2B, however, no obvious expression difference was detected in these NSCLC cell lines. So we chose A549 (adenocarcinoma) and NCI-H520 (squamous carcinoma) cell lines for further investigation. In NSCLC, cancer cells secrete high amounts of proinflammatory and proangiogenic chemokines, such as VEGF, angiopoietin-2, and IL-8, promoting the proliferation and migration in vascular endothelial cells [19]. Here, knockdown of 14-3-3ζ decreased the proliferation and the expressions/secretions of VEGF-A, VEGF-C, angiopoietin-2, and IL-8 mRNAs (Figures 1A, 1B, and Supplementary Figure S2). Moreover, the si-14-3-3ζ-transfected A549 cells recruited less HUVECs in comparison with si-Con group (Figure 1C). Compared with conditioned medium collected from si-Con-transfected NCI-H520 cells, tube formation was reduced dramatically in HUVECs grown in conditioned medium collected from si-14-3-3ζ-transfected cells (Figure 1D). Collectively, we suggested that 14-3-3ζ could be involved in the maintenance of the angiogenic ability in NSCLC cells.

### Effects of 14-3-3ζ on the growth and angiogenesis of NSCLC *in vivo*

**Figure 2:**
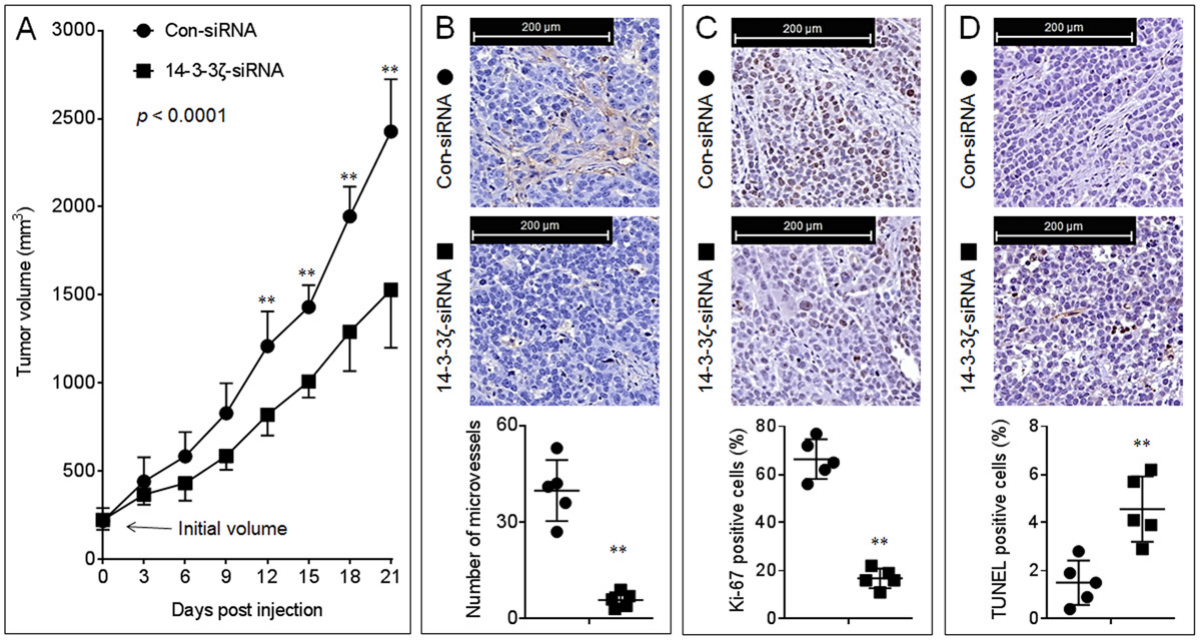
Effects of 14-3-3ζ on the growth and angiogenesis *in vivo* **A.** The volumes of xenografts tumors injected with con-siRNA or 14-3-3ζ-siRNA. (**B.**, top) IHC staining of CD31. (B, bottom) The number of microvessels was quantitated based on the CD31 staining. (**C.**, top) IHC staining of Ki-67. (C, bottom) The percentage of Ki-67 positive cells. (**D.**, top) TUNEL staining. (D, bottom) The percentage of TUNEL positive cells. Note: Each point in (B-D) represented the mean of one xenografts tumor section calculating in 10 high-power fields. ** *p* < 0.01 compared with the xenografts tumors injected with con-siRNA.

To further determine the effects of 14-3-3ζ on the *in vivo* tumor growth and angiogenesis, we conducted an A549 xenografts model. Interestingly, knockdown of 14-3-3ζ significantly inhibited the growth of this xenografts tumor (Figure 2A and Supplementary Figures S3A-S3C). Moreover, compared with xenografts tumor tissues injected with con-siRNA, the 14-3-3ζ-siRNA-injection decreased the formation of intratumoral capillary tubes (as determined by the CD31 positive microvessels, Figure 2B), attenuated the ability of proliferation (as determined by the Ki-67 staining, Figure 2C), and enhanced the apoptosis (as determined by TUNEL staining, Figure 2D). Collectively, these results indicated that, via regulating the proliferation, angiogenesis and apoptosis, 14-3-3ζ stimulated the *in vivo* growth of NSCLC.

### Potential mechanisms involved in the 14-3-3ζ-induced angiogenesis

**Figure 3:**
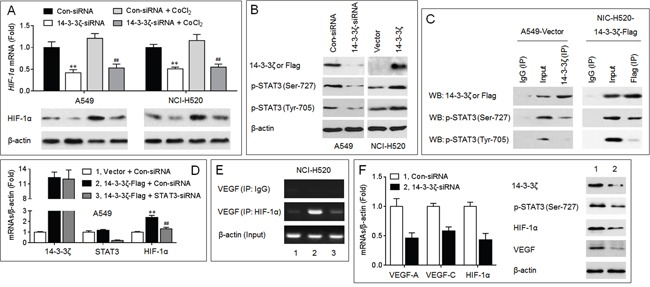
Potential mechanisms involved in the 14-3-3ζ-induced angiogenesis (**A.**, top) qPCR analyses in triplicate of the *HIF-1α* mRNA expression. (A, bottom) Western blots analyses of the HIF-1α protein expression. ** *p* < 0.01 compared with Con-siRNA-transfected group (in the absence of CoCl_2_); ^##^ *p* < 0.01 compared with Con-siRNA-transfected group (in the presence of CoCl_2_). **B.** Western blots analyses of the expressions of 14-3-3ζ (in A549), Flag (in NCI-H520), p-STAT3 (Ser-727), and p-STAT3 (Tyr-705). **C.** Co-IP analyses of the relationship between 14-3-3ζ and p-STAT3. **D.** qPCR analyses in triplicate of the expressions of *14-3-3ζ*, *STAT3*, and *HIF-1α* mRNAs. ** *p* < 0.01 compared with (Vector plus Con-siRNA)-transfected group; ^##^ *p* < 0.01 compared with (Con-siRNA plus 14-3-3ζ-Flag)-transfected group. **E.** ChIP assay analyses of the effects of 14-3-3ζ and STAT3 on the binding of HIF-1α to *VEGF* promoter region. (**F.**, left) qPCR analyses in triplicate of the expressions of *14-3-3ζ*, and *HIF-1α* mRNAs in A549 xenografts tumors. (F, right) Western blots analyses of the expressions of 14-3-3ζ, p-STAT3 (Ser-727), HIF-1α, and VEGF in A549 xenografts tumors. ** *p* < 0.01 compared with xenografts tumors injected with Con-siRNA.

Up to date, a key signaling molecule regulating the angiogenesis in human cancers has been identified, that is the hypoxia-inducible factor-1 alpha (HIF-1α), which transcriptional up-regulates several proangiogenic chemokines, including VEGF [20]. Here, as shown in Figure 3A, knockdown of 14-3-3ζ attenuated the expressions of HIF-1α mRNA and protein in the presence or absence of CoCl_2_ (a hypoxia mimic), indicating that the regulation of HIF-1α by 14-3-3ζ appeared to be through two processes: 1) the transcriptional regulation of HIF-1α *mRNA* (confirmative), and 2) regulating the stabilization of HIF-1α protein (possible). It has been reported that, the phosphorylated-signal transducers and activators of transcription 3 (p-STAT3) can drive the transcription of *HIF-1α* mRNA [21]. Interestingly, 14-3-3ζ can interact with p-STAT3 (Ser-727) and regulates its constitutive activation in human glioblastoma and myeloma cells [22, 23]. Here, in NSCLC cells, overexpression of 14-3-3ζ enhanced the phosphorylation of STAT3 (Ser-727) and STAT3 (Tyr-705); in contrast, knockdown of 14-3-3ζ showed the opposite effects (Figure 3B and Supplementary Figure S4). In addition, 14-3-3ζ formed a complex with p-STAT3 (Ser-727) in NSCLC cells (Figure 3C). Knockdown of STAT3 blocked the 14-3-3ζ-induced increased expression of *HIF-1α* mRNA (Figure 3D), and attenuated the 14-3-3ζ-induced binding of HIF-1α to *VEGF* promoter (Figure 3E). In xenografts model, injection of 14-3-3ζ-siRNA decreased the expressions of p-STAT3, HIF-1α, and VEGF (Figure 3F). Collectively, these results suggested that the STAT3/HIF-1α/VEGF signal pathway might be involved in the 14-3-3ζ-mediated angiogenesis.

### MiR-206 inhibited the 14-3-3ζ/STAT3/HIF-1α/VEGF signaling

**Figure 4:**
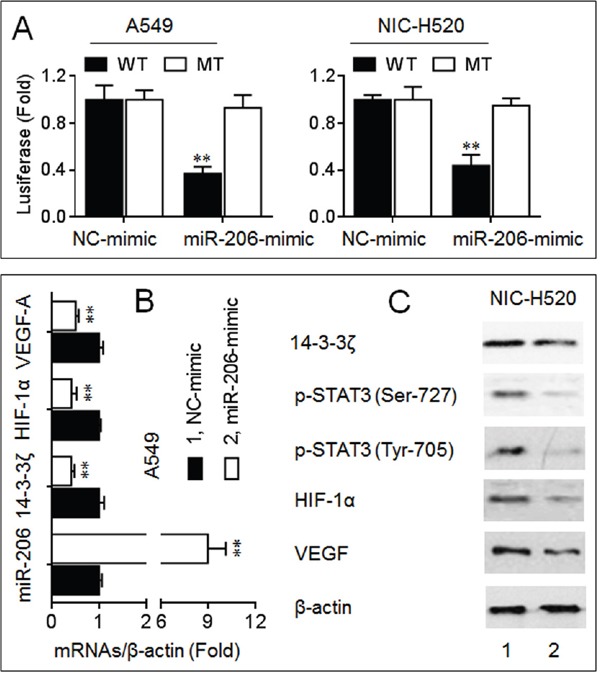
MiR-206 inhibited the 14-3-3ζ/STAT3/HIF-1α/VEGF signaling **A.** Luciferase reporter assay analyses in triplicate of the effects of miR-206 on the 3′-UTR of 14-3-3ζ. **B.** qPCR analyses in triplicate of the expressions of miR-206, *14-3-3ζ*, *HIF-1α*, and *VEGF* mRNAs in A549 cells. **C.** Western blots analyses of the expressions of 14-3-3ζ, p-STAT3 (Ser-727), p-STAT3 (Tyr-705), HIF-1α, and VEGF in NCI-H520 cells. ** *p* < 0.01 compared with NC-mimic-transfected group.

MiR-206 plays an important anti-cancer role via targeting c-Met, Bcl2, cyclinD1, and Sox9 in NSCLC [16–18]. Here, we found a miR-206 binding site in the 3′-UTR of *14-3-3ζ* mRNA (Supplementary Figure S5). A luciferase reporter assay showed that co-transfection with the wild type (WT) constructs and a miR-206-mimic led to a significant decrease of the luciferase activity (Figure 4A). Furthermore, overexpression of miR-206 decreased the endogenous expressions of 14-3-3ζ, p-STAT3, HIF-1α, and VEGF in NSCLC cells (Figure 4B and 4C). So we suggested that miR-206 could function as an important growth/angiogenesis repressor, which exercised its roles possibly via inhibiting the 14-3-3ζ/STAT3/HIF-1α/VEGF pathway in NSCLC cells.

### 14-3-3ζ was involved in the miR-206-inhibited angiogenesis

**Figure 5:**
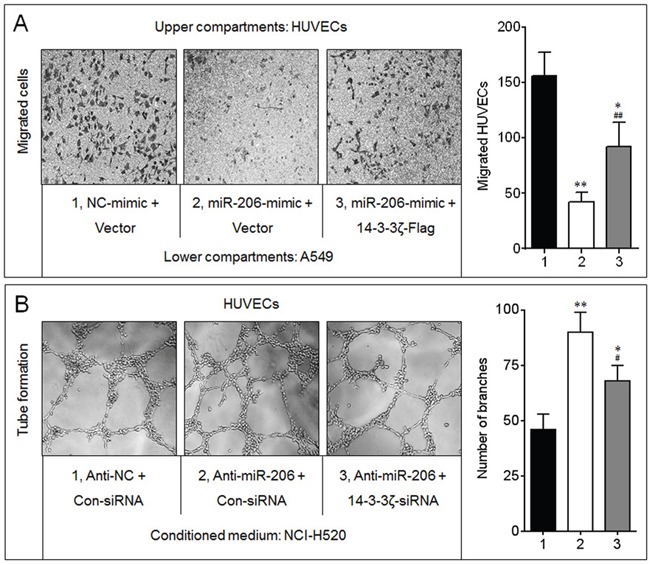
14-3-3ζ was involved in the miR-206-inhibited angiogenesis **A.** Endothelial cells recruitment assay analyses of the effects of miR-206 and 14-3-3ζ on A549-induced recruitment of HUVECs. * *p* < 0.05 and ** *p* < 0.01 compared with the (NC-mimic plus Vector)-transfected group; ^##^ *p* < 0.01 compared with (miR-206-mimic plus Vector)-transfected group. **B.** Capillary tube formation assay analyses of the effects of miR-206 and 14-3-3ζ on angiogenic ability in NCI-H520 cells. * *p* < 0.05 and ** *p* < 0.01 compared with the (Anti-NC plus Con-siRNA)-transfected group; ^##^ *p* < 0.01 compared with (Anti-miR-206 plus Con-siRNA)-transfected group.

In A549 cells, overexpression of miR-206 decreased the recruitment of HUVECs, however, restored the expression of 14-3-3ζ (transfected a 14-3-3ζ expression vector lacking 3′-UTR) partly blocked this phenomenon (Figure 5A). Furthermore, compared with conditioned medium collected from anti-miR-206-transfected NCI-H520 cells, tube formation was reduced dramatically in HUVECs grown in conditioned medium collected from 14-3-3ζ-knockdown cells (Figure 5B). Collectively, these results indicated that the miR-206/14-3-3ζ signaling inhibited angiogenesis in NSCLC cells.

### Effects of miR-206 on the growth, angiogenesis, and 14-3-3ζ/STAT3/HIF-1α/VEGF signal pathway in A549 xenografts

**Figure 6:**
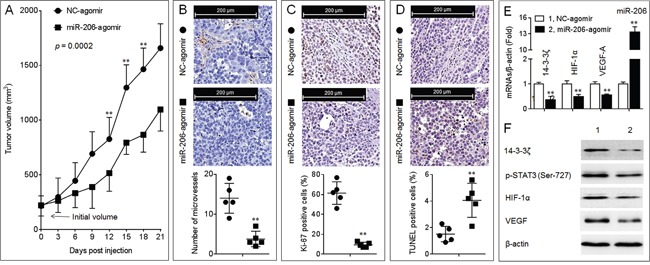
Effects of miR-206 on the growth, angiogenesis, and 14-3-3ζ/STAT3/HIF-1α/VEGF signal pathway in A549 xenografts tumors **A.** The volumes of xenografts tumors injected with NC-agomir or miR-206-agomir. (**B.**, top) IHC staining of CD31. (B, bottom) The number of microvessels was quantitated based on the CD31 staining. (**C.**, top) IHC staining of Ki-67. (C, bottom) The percentage of Ki-67 positive cells. (D, top) TUNEL staining. (D, bottom) The percentage of TUNEL positive cells. Note: Each point in (B-D) represented the mean of one xenografts tumor section calculating in 10 high-power fields. **E.** qPCR analyses in triplicate of the expressions of miR-206, and *14-3-3ζ*, *HIF-1α*, VEGF-A mRNAs in A549 xenografts tumors. **F.** Western blots analyses of the expressions of 14-3-3ζ, p-STAT3 (Ser-727), HIF-1α, and VEGF in A549 xenografts tumors. ** *p* < 0.01 compared with xenografts tumors injected with NC-agomir.

Similar to Figure 2, overexpression of miR-206 significantly inhibited the growth of A549 xenografts tumor (Figure 6A and Supplementary Figures S3D-S3F). Moreover, injection of miRNA-206-agomir decreased the formation of intratumoral capillary tubes (Figure 6B), attenuated the ability of proliferation (Figure 6C), and enhanced the apoptosis (Figure 6D). Further, overexpression of miR-206 attenuated the expressions of 14-3-3ζ, p-STAT3, HIF-1α, and VEGF (Figure 6E and 6F). These results indicated that miR-206 attenuated the *in vivo* growth and angiogenesis of NSCLC, and that the inhibition of 14-3-3ζ/STAT3/HIF-1α/VEGF pathway might be involved in.

### The clinical relationships between miR-206 and 14-3-3ζ in NSCLC

**Figure 7:**
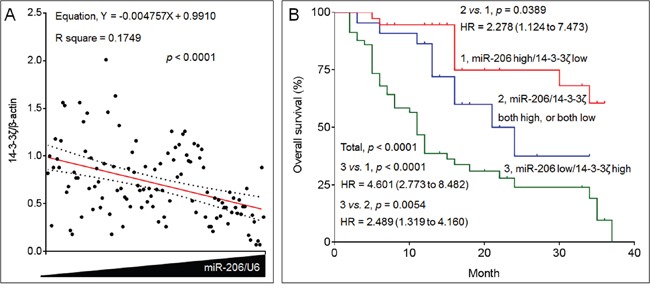
The clinical relationships between miR-206 and 14-3-3ζ in NSCLC **A.** The expressions of miR-206 and 14-3-3ζ mRNAs in 116 NSCLC patients' tissue samples were analyzed via qRT-PCR in triplicate. Each point represented the mean expressions of miR-206 (x axis) and 14-3-3ζ (y axis) in one patient. The correlations between miR-206 and 14-3-3ζ were analyzed. The red line represented the correlation curve and dashed lines represented the 95% confidence intervals. **B.** Kaplan-Meier analyses of the prognostic significances of miR-206 and 14-3-3ζ in these patients.

Studies have showed that either miR-206 or 14-3-3ζ could functions as a predictor for survival in NSCLC [24, 25]. However, the clinical relationships between them remain largely uninvestigated. Here, in NSCLC specimens, with increased expression of miR-206, there were greater decreased expressions of 14-3-3ζ (Figure 7A). So, we finally validated the clinical significance of the combination of miR-206 and 14-3-3ζ in NSCLC. The cohort of 116 NSCLC patients were divided into “miR-206 high/14-3-3ζ low”, “miR-206/14-3-3ζ both high, or both low”, and “miR-206 low/14-3-3ζ high” groups. Kaplan-Meier survival analysis also showed that patients in “miR-206 low/14-3-3ζ high” group had the worst survival than those in “miR-206 high/14-3-3ζ low” group (Figure 7B).

## DISCUSSION

During angiogenesis, a number of angiogenic molecules can promote the tumor cells to recruit the vascular endothelial cells and form the intratumoral capillaries [19]. Of these, the most critical appears to be the VEGF, which does not affect NSCLC cell proliferation, but significantly induced the secretion of a variety of angiogenic factors, including angiopoietin-2 and IL-8 [26]. Interestingly, in NSCLC cells, VEGF also activates the phosphatidylinositol-3-kinase, extracellular signal-regulated kinase 1/2, and STAT3 signalling pathways, which further stimulate the NSCLC cells to secret VEGF [26]. Here we found that the 14-3-3ζ elevated the autocrine of VEGF, angiopoietin-2, and IL-8, and enhanced the phosphorylation of STAT3, suggesting that 14-3-3ζ might be a positive regulator of this autocrine feed-forward loop.

Deficit of oxygen availability within tumor microenvironment directly regulates the HIF-1α signal [27], however, the activation of HIF-1α is not only induced by hypoxia, but also via various kinases cascade, for example, STAT3 [21]. Indeed, the phosphorylation of STAT3 can drive the transcription of *HIF-1α* mRNA [21]. Furthermore, either the activation of HIF-1α or STAT3 can transcriptionally elevate the expression of *VEGF* [28, 29]. In our present study, 14-3-3ζ bond to p-STAT3 (Ser-727) and increased its activation. In addition, 14-3-3ζ also improved the phosphorylation of STAT3 (Tyr705). These results indicated that 14-3-3ζ might be an important trigger, which initiated the STAT3/HIF-1α/VEGF feed-back loop.

In human genome, miR-206 is similar to miR-1 in terms of expression and function, but its sequence differs from the miR-1 sequence by four nucleotides [30]. Indeed, miR-1-1/miR-133a-2, miR-1-2/miR-133a-1, and miR-206/miR-133b form clusters in three different chromosomal regions [30]. It has been showed that, miR-1, miR-133a, miR-133b and miR-206 are inhibited in various types of cancers, including NSCLC [31–34]. Here, we did not find the miR-133a/b binding sites in the 3′-UTR of *14-3-3ζ*; however, there were miR-1/206 binding sites in the 3′-UTR of *14-3-3ζ*, *14-3-3θ/τ* and *14-3-3ε*. So the net-work between miR-1/206 and 14-3-3 protein family is very complex. Our present study expanded our understanding of miR-206-caused anti-tumor effects in lung cancer. However, it is likely that via targeting the 14-3-3 protein family members (ζ, θ/τ, and ε), miR-206 and/or miR-1 have additional functions. So, future studies should be performed to determine whether miR-1 plays the same role in NSCLC, and whether the anti-tumor effects of miR-1 and/or miR-206 were mediated via targeting the other 14-3-3 protein family members (θ/τ, and ε).

## MATERIALS AND METHODS

### Cell culture and transfection

The human NSCLC cell lines (A549 and NCI-H520) and human umbilical vein endothelial cell line (HUVECs), were obtained from Institute of Biochemistry and Cell Biology, Shanghai Institutes for Biological Sciences, Chinese Academy of Sciences. These cells were identified by China Center for Type Culture Collection (Wuhan, China). Cells were maintained in a 37°C humidified incubator with 5% CO_2_. NCI-H520 cells were cultured in Dulbecco's Modified Eagle Medium (DMEM, Life Technologies/Gibco, Grand Island, NY), A549 cells were cultured in RPMI-1640 medium (Life Technologies/Gibco), and HUVECs were cultured in ECM medium (Invitrogen, Carlsbad, USA). The mediums were supplemented with 10% fetal bovine serum, 100 U/ml penicillin, 100 μg/ml streptomycin (Gibco), 100 μg/ml heparin, and 30 μg/ml ECs growth supplement (for HUVECs, Sigma-Aldrich, MO, USA). Phenol red was added into the medium to reflect the pH. A mycoplasma stain assay Kit (Beyotime Co. Ltd, Haimeng, China) was used for mycoplasma testing. For miRNA transfection, the miR-206- or NC-mimics (Supplementary Table S1) were chemically synthesized by RiboBio Co (Guangzhou, China), and the transfection was conducted as we described previously [35]. For overexpression or knockdown of 14-3-3ζ, the pcDNA-3.1-14-3-3ζ-Flag plasmid that overexpressed both 14-3-3ζ and Flag was created by inserting the coding sequences of 14-3-3ζ (YWHAZ, 738 bp) into pcDNA3.1 plasmid, followed by adding a Flag tag at its N-terminal (Generay Biotech Co. Ltd, Shanghai, China), the commercial specific siRNAs directed against human 14-3-3ζ or STAT3 were purchased from Santa Cruz Biotechnology (http://datasheets.scbt.com/sc-29583.pdf and http://datasheets.scbt.com/sc-29493.pdf). The protocol of transfection was as we described previously [36].

### Animals, xenografts, and intratumoral injection assay

This study was approved by Nanjing Medical University Institutional Animal Care and Use Committee, and animals were treated humanely and with regard for alleviation of suffering. The obtaining and keeping of the BALB/c nude mice were as we described previously [36]. For xenograft study, 2×10^6^ A549 cells in 100 μl matrigel were injected subcutaneously into the right armpit of the mice (5 mice per group) for 3 weeks. To determine the effects of miR-206 and/or 14-3-3ζ on the *in vivo* angiogenesis of NSCLC, we performed the intratumoral injection assay. Briefly, 50 μl of miRNA-agomir (NC-agomir or miR-206-agomir, 60 nM, RiboBio Co) or 100 μl of siRNA (si-Con or si-14-3-3ζ, 100 nM) were intratumoral injections every 3 days. Tumors were measured every 3 days and their volumes were calculated using the formula: V= ½ (width^2^ × length). After 21 days, the mice were sacrificed, and tumor tissues were removed for further investigation.

### Ethics statement and patients

This study was approved by Medical Ethics Committee of Nanjing Medical University, and the written informed consents were obtained from each patient. A total of 116 patients who underwent surgery for histologically verified NSCLC between Mar 2013 and Oct 2015 were enrolled. None of them received any preoperative anticancer treatment prior to sample collection. The clinicopathologic characteristics of the patients are shown in Supplementary Table S1. All patients were followed after surgical treatment until Jun 2016. Patients were monitored every 3 months, and the CT was performed when tumor recurrence was suspected.

**For other common functional assays:** please see the SUPPLEMENTARY MATERIALS AND METHODS section.

## SUPPLEMENTARY DATA, SUPPLEMENTARY TABLES AND FIGURES



## Abbreviations

Ang-2: angiopoietin-2
HUVECs: human umbilical vein endothelial cells
HIF-1α: hypoxia-inducible factor-1 alpha
miRNAs: microRNAs
NSCLC: non-small cell lung cancer
STAT3: signal transducers and activators of transcription 3
TUNEL: transferase-mediated deoxyuridine triphosphate-biotin nick end labeling
VEGF: vascular endothelial growth factor
