# Supplementary material for: MicroRNA-206 attenuates the growth and angiogenesis in non-small cell lung cancer cells by blocking the 14-3-3ζ/STAT3/HIF-1α/VEGF signaling
Source: Oncotarget. 2016 Oct 28;7(48):79805–13. doi: 10.18632/oncotarget.12972 (PMC5346752; doi:10.18632/oncotarget.12972)
Supplement: Supplementary file 1 [file oncotarget-07-79805-s001.pdf]

MicroRNA-206 attenuates the growth and angiogenesis in non-small cell lung cancer cells by blocking the 14-3-3ζ/STAT3/HIF-1α/VEGF signaling

SUPPLEMENTARY DATA

Growth kinetics

For the determination of growth kinetics, 1×10^5 cells were seeded in six-well plates, followed by conventional cultured for 24, 48, or 72 h, respectively. At each time, the cells were collected and counted in triplicate using a hemocytometer under a microscope. The doubling time was calculated using the formula: $Td = \Delta t \times \log2/ (\log Nt - \log N0)$ as we described previously [1].

Quantitative real-time polymerase chain reaction (qRT-PCR)

Primers used were listed in Supplementary Table S2. Total RNA was isolated using Trizol (Invitrogen) according to the manufacturer's recommendations. Then total RNA (2 μg) was transcribed into cDNA using AMV Reverse Transcriptase (Promega, Madison, USA). qRT-PCR was performed using the Applied Biosystems 7300HT machine (Applied Biosystems, CA, USA) and MaximaTM SYBR Green/ROX qPCR Master Mix (Fermentas, MA, USA). The PCR reaction was evaluated using melting curve analysis. β-actin was amplified to ensure cDNA integrity and to normalize expression. Fold changes in expression of each gene were calculated by a comparative threshold cycle (Ct) method using the formula $2^{-(\Delta\Delta Ct)}$.

Enzyme-linked immunosorbent assay (ELISA)

A total of 1×10^5 cells were seeded in 6-well plates. After treatments, the mediums were collected, cleared by centrifugation. ELISA was performed using the human VEGF, angiopoietin-2, or CXC chemokine ligand 8/IL-8 Quantikine kit (R&D Systems, MN, USA) according to the manufacturer's protocol. Recombinant human VEGF, angiopoietin-2, or IL-8 was used for calibration. The absorbance at 450 nm was measured with a multimode microplate reader (Tecan, Männedorf, Switzerland).

Western blots

Total protein was extracted by lysing cells in RIPA buffer (Beyotime Co. Ltd). Then the protein concentrations were measured with the BCA kit (Beyotime Co. Ltd). Afterwards, proteins (20 μg) were separated by 10% sodium dodecyl sulfate-polyacrylamide gel electrophoresis followed by transferring to polyvinylidene fluoride membranes (Millipore, Billerica, USA). After blocking with 10% non-fat milk in TBST for 1 h at the room temperature, the membranes were incubated with the primary antibody (Supplementary Table S3) at 4°C overnight. Then, the membranes were incubated with horseradish peroxidase-conjugated secondary antibodies (1: 2000, Beyotime Co. Ltd) for 1 h at the room temperature. The immune complexes were detected by the enhanced chemiluminescence (Cell Signaling Technology, Beverly, MA, USA). Densitometric analysis was conducted via an Image-Pro-Plus 6.0 software (Media Cybernetics, Georgia, USA).

HUVECs recruitment assay

As we described previously [1], HUVECs (5×10^4) were seeded in the upper compartments of the chambers, [24-well Boyden chambers with 8-μm pore size polycarbonate membranes (Corning, NY, USA)], and NSCLC cells were placed in the lower compartments. The co-cultured tumor cells were pre-transfected by vector, 14-3-3ζ-Flag, Con-siRNA, 14-3-3ζ-siRNA, NC-mimic, miR-206-mimic, anti-Con, or anti-miR-206, respectively for 12 h and refreshed with DMEM before the recruitment experiments. The chambers were incubated at 37 °C for 24 h. Cells migrating through the filter to the lower surface were fixed with 4% paraformaldehyde and then stained with 0.1% crystal violet. Migrated cells were viewed and photographed under a phase-contrast microscope (Olympus, Tokyo, Japan), and counted by Image-Pro-Plus 6.0 software in five randomly chosen fields.

Capillary tube formation assay

For the tube formation assay, 1×10^4 HUVECs were seeded in a 24-well plate on matrigel that had polymerized for 30 min at 37 °C. After then, such cells were incubated in the conditioned mediums collected from vector-, 14-3-3ζ-Flag-, Con-siRNA-, 14-3-3ζ-siRNA-, NC-mimic-, miR-206-mimic-, anti-Con-, or anti-miR-206-transfected NSCLC cells for another 6 h, respectively. Capillary morphogenesis was evaluated by using an inverted microscope (Olympus), and quantitated in five randomly chosen fields as we described previously [1].

Co-immunoprecipitation (Co-IP)

Cells were extracted for 30 min with immunoprecipitation lysis buffer (Beyotime Co. Ltd). After centrifugation of the preparations, the supernatants were (total proteins) concentrations were measured with the BCA kit (Beyotime Co. Ltd). Then 100 μg proteins were incubated with 14-3-3ζ-Flag antibody at 4°C overnight. Then the protein-antibody complex were incubated with IgA plus IgG sepharose beads (Beyotime Co. Ltd) at 4°C for another 12 h. After then, the supernatants were removed and the beads were washed for three times, resuspended in the SDS sample buffer (Beyotime Co. Ltd), and boiled to remove protein from the beads. Then, such protein samples were analyzed by Western blots with STAT3 (Ser-727) or STAT3 (Tyr-705) antibody. Densitometric analysis was conducted via an Image-Pro-Plus 6.0 software (Media Cybernetics).

Luciferase reporter assay

The pGL3-14-3-3ζ-3'-UTR (wild type, WT; or mutant, MT, Figure S5)-Luc constructs were synthesized by Shuntian Bio Co. (Shanghai, China). The plasmid phRL-tk containing the Renilla luciferase gene was purchased from Promega. Briefly, cells were plated in 24-well culture dishes. When cells proliferated to 60 to 80% confluence, Con-mimics or miR-206-mimics was co-transfected with the respective reporter construct, by using Lipofecamine 2000 reagent (Invitrogen) according to the manufacturer's protocol. The cells were lysed with passive lysis buffer (Promega), and the lysates were analyzed immediately with a 96-well plate luminometer (Berthold Detection System, Pforzheim, Germany). The amounts of luciferase and Renilla luciferase were measured with the Dual-Luciferase Reporter Assay System Kit (Promega) following the manufacturer's instructions.

Chromatin-Immunoprecipitation (ChIP) assay

NSCLC cells were transfected by Con-siRNA or 14-3-3ζ-siRNA for 12 h. Then, to crosslink proteins to DNA, such cells were treated with 1% formaldehyde in PBS by gentle agitation at room temperature for 10 min. Next, cells were washed, resuspended in lysis buffer, and sonicated on ice for 15 sec × 10 times at a 25% output in an EpiShear probe sonicator (Active Motif, Carlsbad, CA, USA). For the immunoprecipitation of protein-DNA complexes, 5 μl of specific magnetic Dynal bead (Invitrogen)-coupled antibody against HIF-1α or an isotype IgG (an negative control) was added at 4°C overnight, followed by a centrifugation (3000 round/min × 1 min). Then, the immunoprecipitated DNA and input DNA (total DNA) were cleaned by RNase A (0.2 mg/ml) and proteinase K (2 mg/ml, Beyotime Co. Ltd) before phenol/chloroform-purification. The specific sequences from immunoprecipitated and input DNA were determined by PCR analysis. Amplifications of the VEGF gene sequences among the pull of DNA were performed with specific primers flanking from -1041 to -750 in the VEGF promoter upstream regions (Supplementary Table S1). The PCR conditions for were 94 °C for 1 min; 35 cycles of 94 °C for 45 sec, 60 °C for 45 sec, and 72 °C for 45 sec; followed by 72 °C for 10 min, and cooling at 4 °C. The PCR products were then analysed by an agarose gel electrophoresis.

Immunohistochemistry (IHC)

Sections mounted on silanized slides were dewaxed in xylene; dehydrated in ethanol; boiled in 0.01 M citrate buffer, pH 6.0, for 20 min in a microwave oven; and then incubated with 3% hydrogen peroxide for 5 min. After washing with PBS, sections were incubated in 10% normal bovine serum albumin for 5 min, followed by incubation with a rabbit-anti Ki-67 (1: 50), or rabbit-anti CD31 (1: 50) antibody at 4°C overnight. Then the slides were incubated with an anti-rabbit horseradish peroxidase-conjugated secondary antibody (1: 300, Beyotime Co. Ltd) at room temperature for another 30 min. The staining was visualized using diaminobenzadine, and the sections were counterstained with hematoxylin, dehydrated, cleared, mounted, and photographed under a pannaramic-scan digital slice scanning system (3DHISTECH Co. Ltd, Budapest, Hungary). The graphs were analyzed via Image-Pro-Plus 6.0 software by two independent researchers who were blinded regarding the experiments' details. The protocol of IHC Quick-score and the inclusion criteria of intratumoral microvessels were according to our previous study [2].

Tunnel staining

Following the manufacturer's instruction (Roche Diagnostics Ltd, Shanghai, China), sections were pretreatment for dewaxing and dehydration as previously descripted in IHC, and then incubated with proteinase K (10 μg/ml, Beyotime Co. Ltd) for 20 min at 37 °C. After washing with PBS, sections were incubated with TUNEL reaction mixture (negative control without terminal transferase) at 37 °C in for 60 min in dark. Then samples were incubated with Converter-POD at 37 °C for 30 min and visualized using diaminobenzadine under microscope after wash in PBS. Finally, the sections were counterstained with hematoxylin, dehydrated, cleared, mounted, and analyzed as mentioned in IHC.

Statistical analysis

Data sets were compared using Graphpad 6.0 (GraphPad Software, Inc, CA, USA) and/or IBM SPSS software v20.0 (IBM Corp, Armonk, USA). Data were presented as the means ± SD. The difference between two groups was analyzed using a two-tailed student's t test. For the repeated measures data, a one- or two-way ANOVA followed by a Sidak's multiple comparisons test was used. The p values <0.05 were considered statistically significant.

REFERENCES

1. Li, Y, Xu, Y, Ling, M, Yang, Y, Wang, S, Li, Z, et al., mot-2-Mediated cross talk between nuclear factor-B and p53 is involved in arsenite-induced tumorigenesis of human embryo lung fibroblast cells. Environmental health perspectives. 118 (2010) 936-942.
2. Shen, J, Jiang, F, Yang, Y, Huang, G, Pu, F, Liu, Q, et al., 14-3-3eta, a novel growth-promoting factor in hepatocellular carcinoma, not only in tumor but also in intratumoral vessels. Journal of hepatology. 2016, 65:953-962.

SUPPLEMENTARY TABLES AND FIGURES

Supplementary Table S1: Association between the expression of miR-206/14-3-ζ and clinicopathological characteristics in 116 NSCLC patients

Characteristics	Case	miR-206 expression		*p*	14-3-ζ expression		*p*
		High (58)	Low (58)		High (58)	Low (58)	
Age				0.3331			0.1981
≤ 60 years	67	31	36		37	30	
> 60 years	49	27	22		21	28	
Gender							
Male	83	40	43	0.4531	44	39	0.2991
Female	33	18	15		14	19	
Tumor size				0.002			0.0001
≤ 3 cm	70	43	27		22	48	
> 3cm	46	15	31		36	10	
Differentiation				0.0643			0.0219
Well-moderate	31	20	11		10	21	
Poor	85	38	47		48	37	
TNM stage				0.0027			0.0001
I-II	77	46	31		29	48	
III	39	12	27		29	10	

The expressions of miR-206 and 14-3-3ζ mRNAs in 116 NSCLC tissue samples were determined in triplicate by qRT-PCR. The boundary (cut-off) was defined as the median values as described previously [2].

Supplementary Table S2: Primers and miRNA transfection reagents used in this study

Names	Primers (5'-3')
14-3-ζ	GCCTGTGAGCAGCGAGATCC (F)<br>AGCATGGATGACAAATGGTC (R)
angiopoietin-2	AGCAGATTTTGGATCAGACCAG (F)<br>GCTCCTTCATGGACTGTAGCTG (R)
HIF-1α	TTTGGCAGCAACGACA (F)<br>AGCGGTGGGTAATGGA (R)
HIF-1α (ChIP)	CAGGAACAAGGGCCTCTGTCT (F)<br>TGTCCCTCTGACAATGTGCCATC (R)
IL-8	ATGACTTCCAAGCTGGCCGTG (F)<br>CTCAGCCCTCTTCAAAAACTTCTC (R)
VEGF-A	CGAACGTACTTGCAGATGTG (F)<br>CTGTTCTGTCGATGGTGATG (R)
VEGF-C	CTGCCGATGCATGTCTAAAC (F)<br>CTTGTTCGCTGCCTGACAC (R)
miR-206	CTCAGCGGCTGTCGTGGACTGCGCGCTGCCGCTGAGCCACACAC (for reverse transcriptase)<br>GGCGGTGGAATGTAAGGAAG (F)<br>GGCTGTCGTGGACTGCG (R)
miR-206-mimic	UGGAAGUAAGGAAGUGUGUGG (S)<br>ACACACUUCCUUACAUUCCAUU (A)
anti-miR-206	CCACACACUUCCUUACAUUCCA

Supplementary Table S3: Antibodies used in this study

Names	Companies	Dilutions
14-3-3ζ	Cell Signaling Technology	1: 1000 (WB), 1: 100 (IP)
CD-31	Santa Cruz Biotechnology	1: 50 (IHC)
Flag	Beyotime Co. Ltd	1: 1000 (WB), 1: 200 (IP)
HIF-1α	Novus	1: 1000 (WB), 1: 100 (IP)
Ki-67	Abcam	1: 100 (IHC)
p-STAT3 (Ser-727)	Cell Signaling Technology	1: 500 (WB)
p-STAT3 (Tyr-705)	Cell Signaling Technology	1: 500 (WB)
STAT3	Cell Signaling Technology	1: 1000 (WB)
VEGF	R&D Systems	1: 500 (WB)

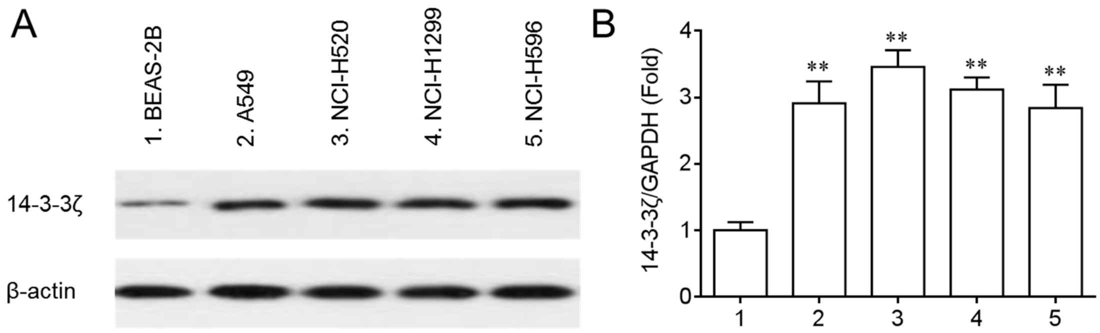

Supplementary Figure S1: A. Western blots analyses of the expression of 14-3-3ζ in (1) human normal bronchial epithelial BEAS-2B cells, (2) adenocarcinoma A549 cells, (3) squamous cell carcinoma NCI-H520 cells, (4) large cell carcinoma NCI-H1299 cells, and (5) adenosquamous carcinoma NCI-H596 cells. **B.** Summary of 14-3-3ζ expression from three experiments. The expression levels of 14-3-3ζ and β-actin were determined as in (A) and quantified by densitometry. The ratio of 14-3-3ζ/β-actin in BEAS-2B cells was defined as 1 fold. ** $p < 0.01$ compared with the group 1.

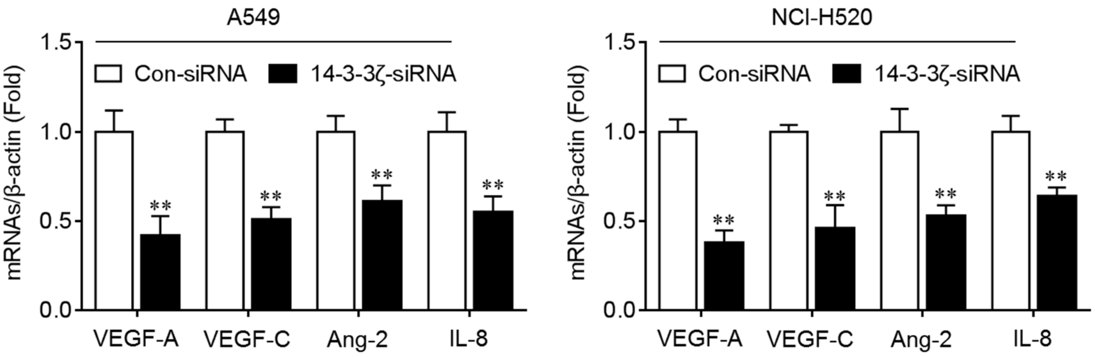

Supplementary Figure S2: qRT-PCR analyses in triplicate of the *VEGF-A, VEGF-C, angiopoietin-2* (Ang-2), and *IL-8* mRNAs in Con-siRNA- or 14-3-3ζ-siRNA transfected NSCLC cells. The ratios of mRNAs/β-actin in Con-siRNA-transfected cells were defined as 1 fold. ** $p < 0.01$ compared with the Con-siRNA-transfected cells.

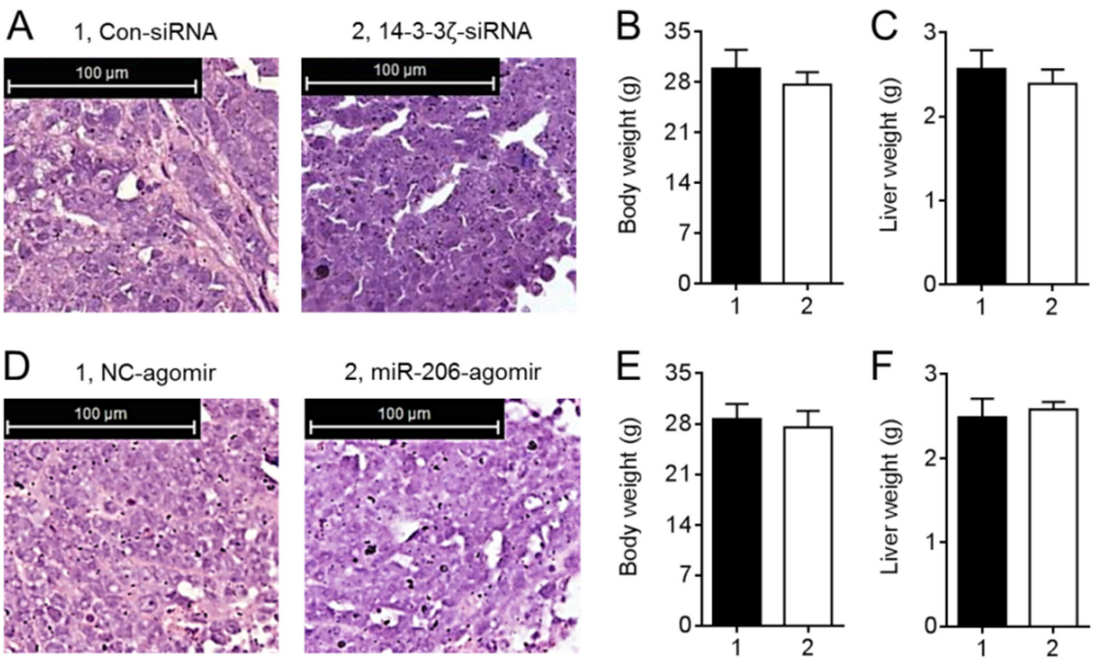

Supplementary Figure S3: A and **D.** H&E staining. **B** and **E.** Body weight and **C** and **F.** Liver weight (5 mice per group). The body weight and liver weight were similar among groups.

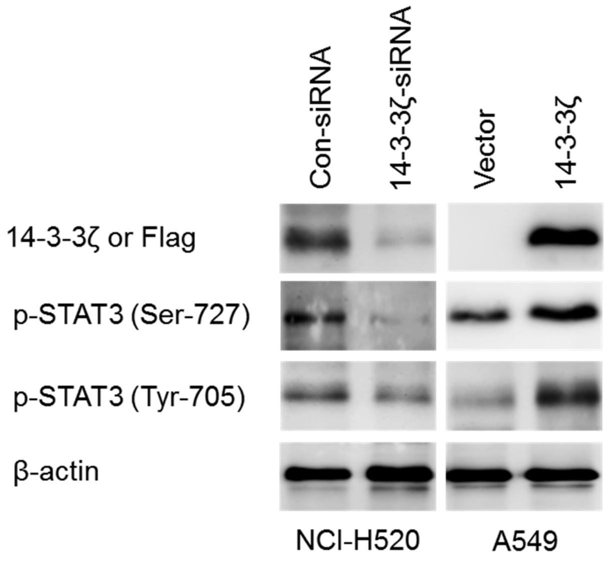

Supplementary Figure S4: Western blots analyses of the expressions of 14-3-3ζ (in NCI-H520), Flag (in A549), p-STAT3 (Ser-727), and p-STAT3 (Tyr-705).

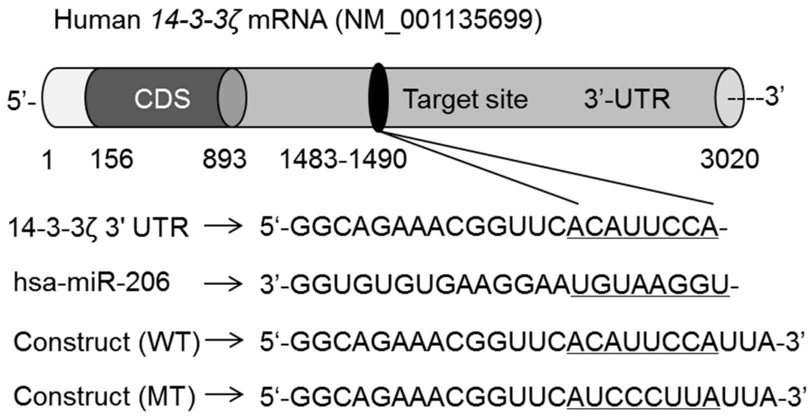

Supplementary Figure S5: The target sequences of miR-206 in the 3'-UTR of *14-3-3ζ* mRNA.
